# Microglia P2X4 receptor contributes to central sensitization following recurrent nitroglycerin stimulation

**DOI:** 10.1186/s12974-018-1285-3

**Published:** 2018-08-30

**Authors:** Ting Long, Wei He, Qi Pan, Shanshan Zhang, Yixin Zhang, Chaoyang Liu, Qing Liu, Guangcheng Qin, Lixue Chen, Jiying Zhou

**Affiliations:** 1 Department of Neurology, The First Affiliated Hospital of Chongqing Medical University, 1st Youyi Road, Yuzhong District, Chongqing, 400016 China; 2 Laboratory Research Center, The First Affiliated Hospital of Chongqing Medical University, Chongqing, China

**Keywords:** P2X4R, Microglia, Migraine, Nitroglycerin, Animal model

## Abstract

**Background:**

The mechanism underlying migraine chronification remains unclear. Central sensitization may account for this progression. The microglia P2X4 receptor (P2X4R) plays a pivotal role in the central sensitization of inflammatory and neuropathic pain, but there is no information about P2X4R in migraine. Therefore, the aim of this study was to identify the precise role of microglia P2X4R in chronic migraine (CM).

**Methods:**

We used an animal model with recurrent intermittent administration of nitroglycerin (NTG), which closely mimics CM. NTG-induced basal and acute mechanical hypersensitivity were evaluated using the von Frey filament test. Then, we detected Iba1 immunoreactivity (Iba1-IR) and P2X4R expression in the trigeminal nucleus caudalis (TNC). To understand the effect of microglia and P2X4R on central sensitization of CM, we examined whether minocycline, an inhibitor of microglia activation, and 5-BDBD, a P2X4R antagonist, altered NTG-induced mechanical hyperalgesia. In addition, we also evaluated the effect of 5-BDBD on c-Fos and calcitonin gene-related peptide (CGRP) expression within the TNC.

**Results:**

Chronic intermittent administration of NTG resulted in acute and chronic basal mechanical hyperalgesia, accompanied with microglia activation and upregulation of P2X4R expression. Minocycline significantly decreased basal pain hypersensitivity but did not alter acute NTG-induced hyperalgesia. Minocycline also reduced microglia activation. 5-BDBD completely blocked the basal and acute hyperalgesia induced by NTG. This effect was associated with a significant inhibition of the NTG-induced increase in c-Fos protein and CGRP release in the TNC.

**Conclusions:**

Our results indicate that blocking microglia activation may have an effect on the prevention of migraine chronification. Moreover, we speculate that the P2X4R may be implicated in the microglia-neuronal signal in the TNC, which contributes to the central sensitization of CM.

**Electronic supplementary material:**

The online version of this article (10.1186/s12974-018-1285-3) contains supplementary material, which is available to authorized users.

## Background

Migraine is a complex, severe neurological disease that has been intensively researched. Each year, 2.5% of episodic migraine patients convert into chronic migraine (CM) [1]. With chronification, there are structural, physiologic, and biochemical alterations in the brain that lead to an increase in the frequency of headache and a poor response to therapy [2]. The mechanism of migraine chronification is still unclear. One of the leading theories is the central sensitization hypothesis, which proposes that there is an increase in the excitability of central neurons in the trigeminal nociceptive pathway, principally the trigeminal nucleus caudalis (TNC) [3].

Neurons, as the center of pain information processing, transmission, and integration, have been the focus of central sensitization. Therefore, many “classic” analgesic drugs primarily target neurons. But increasing evidence suggests that activation of microglia directly or indirectly influences the establishment of neuronal hyperexcitability, which plays an important role in the development or maintenance of chronic pain states, including migraine [4–6]. Tsubasa Takizawa and colleagues noted that multiple cortical spreading depression (CSD), considered to be associated with migraine aura, could lead to microglia activation [5]. Meanwhile, a study indicated that repeated inflammatory soup (IS) infusions stimulated the dura mater, which also can lead to microglia activation [7]. Therefore, a better understanding of the molecular cross-talk mechanism between TNC neurons and surrounding microglia could help lead to a profound understanding of migraine chronification.

Adenosine triphosphate (ATP), as an algogenic substance, mediates either the activation or the sensitization of nociceptive neurons via ATP receptors (ionotropic P2X-purinoceptor or metabotropic P2Y-purinoceptor). The potential involvement of the purinergic signaling in the pathophysiology of migraine was put forward more than 30 years ago [8]. Since then, the roles of different types of purinergic receptors have been examined in cellular and animal models of migraine, among which, the role of P2X3R is the most widely studied [9–11]. Moreover, these studies mainly focused on the peripheral sensitization mechanism of migraine or CSD, whereas few focused on central sensitization mechanism.

Activated microglia induce or enhance expression of various purinergic P2 receptor. P2X4R belongs to the P2X family of receptors, which are mainly expressed in microglia. Extensive lines of evidence indicate that P2X4R-positive microglia play a pivotal role in the mechanism of neuropathic pain. Inhibiting the expression and function of P2X4R suppresses the central sensitization of neuropathic pain [12]. However, the role of P2X4R has not been examined in any animal model of migraine.

Therefore, the objective of this study was to investigate the role of microglia and P2X4R in CM. A mouse model of CM was developed using chronic intermittent nitroglycerin (NTG) injection, and then, the effect of the pharmacological blockade of microglia activation and P2X4R on NTG-induced mechanical hyperalgesia, c-Fos, and calcitonin gene-related peptide (CGRP) expression in the TNC was examined.

## Methods

### Animals

Male C57BL/6 mice (weighting 18–20 g, aged 8–10 weeks) were provided by the Experimental Animal Center of Chongqing Medical University (Chongqing, China). All procedures followed the National Institutes of Health Guidelines for the Care and Use of Laboratory Animals. Mice were maintained on a 12-h light/dark cycle under standard laboratory conditions with enough food and water. Experiments were carried out between 9:00 and 15:00. All animals were acclimatized for 1 week before experimental procedures and randomly assigned to experimental groups. Randomized allocation was designed with Excel Software. The procedure was as follows: (1) C67BL/6 mice weighting 18–20 g were selected then numbered. (2) The randomization numbers were created using the “rand()” function of Excel software and sorted by incremental method. (3) Then, according to the order, each 6–8 numbers are divided into different groups.

### Drugs and treatments

NTG (Beijing Regent, China) was prepared from a stock solution of 5.0 mg/ml dissolved in 30% alcohol, 30% propylene glycol, and water. NTG was diluted at 1 mg/ml prior to the start of the experiment. The vehicle control was 0.9% saline because there was no significant difference in mechanical thresholds between 0.9% saline and the solution in which NTG was dissolved (6% propylene glycol, 6% alcohol, 0.9% saline). Animals received intraperitoneal (i.p.) injections of 10 mg/kg NTG or vehicle every other day for 9 days (i.e., days 1, 3, 5, 7, and 9) [13].

To understand the effect of microglia and P2X4R on NTG-induced hyperalgesia, we treated the animals with the microglia inhibitor minocycline (MedChemExpress/MCE, American) or the P2X4R-selective antagonist 5-BDBD (Sigma-Aldrich, American). Minocycline and 5-BDBD were dissolved in DMSO solution, which was used as the vehicle control. Minocycline/vehicle or 5-BDBD/vehicle was administered immediately prior to the administration of NTG/saline [14, 15]. The treatment of the mice on testing days was as follows: mice were habituated to the test room, followed by a baseline measurement of mechanical thresholds 15–20 min later and an administration of minocycline (30 mg/kg, i.p.), 5-BDBD (28 mg/kg, i.p.), or vehicle; then, an injection of NTG/saline was given, and 2 h later, acute mechanical responses were tested. This procedure was repeated every other day for 9 days.

### Sensory sensitivity testing

All animal behavior testing occurred in light conditions, between 09:00 and 15:00. For the determination of mechanical sensitivity, the threshold for responses to punctuate mechanical stimuli (mechanical hyperalgesia) was tested according to the up-and-down method. The plantar surface of the animal hind paw was stimulated with a series of eight von Frey filaments (bending force ranging from 0.01 to 2 g) from the underside of the mesh stand. The 0.4-g von Frey filament was tested first. A response was defined as a withdrawal, shaking, or licking of the paw. In the absence of a response, a heavier filament (up) was used, and in the presence of a response, a lighter filament (down) was tested. Each filament was applied to the skin for 3 s at 1-min intervals. This pattern was followed for a maximum of four filaments following the first response. The evaluating experimenters were blind to the experimental groups.

### Quantitative real-time polymerase chain reaction (qRT-PCR)

Mice were euthanized, and the TNC was dissected at 12 h after last NTG injection for qRT-PCR experiments. The tissues were immediately stored in liquid nitrogen. Total RNA was extracted as previously described [16] using an RNAiso Plus reagent (TaKaRa, Dalian), and RNA was quantified spectrophotometrically with NanoDrop (Thermo, USA). Then, cDNA was synthesized using the PrimeScript™ RT Reagent Kit (Takara). P2X4R (p2rx4) mRNA expression are analyzed. Specific primers were obtained from Sangon Biotech (Shanghai, China). The sequences of primers are described as follows: P2X4: 5′-TCG TGT GGG AAA AGG GCT AC-3′ (forward), 5′-GTC TGG TTC ACG GTG ACG AT-3′ (reverse); GAPDH: 5′-ATG ACT CTA CCC ACG GCA AGC T-3′ (forward), 5′-GGA TGC AGG GAT GAT GTT CT-3′ (reverse). GAPDH mRNA was used as an endogenous control for normalization. Quantitative real-time PCR was performed with SYBR® Premix Ex Taq™ II (Takara) with a CFX96 Touch thermocycler (Bio-Rad, USA) according to the instructions. All fluorescence data were processed by a post-PCR data analysis software program. The ΔΔCq method was used to investigate the differences in the gene expression levels.

### Western blot analysis

For P2X4R protein detection, TNC was collected 12 h after different NTG injection times. Until measurements, the samples were stored at − 80 °C. Tissues were sonicated in an ice-cold RIPA lysis buffer (Beyotime, China) containing with phenylmethylsulphonyl fluoride (PMSF, Beyotime, China) at 4 °C for 1 h. Protein concentration was measured according to the BCA protein assay method with a BCA Protein Assay Kit (Beyotime, China). Equal amounts of protein (40 μg) were loaded on an SDS-PAGE gel (Beyotime, China), electrophoresed, and transferred to PDVF membranes. Following the transfer, membranes were blocked for 2 h at room temperature in TBST containing 5% non-fat milk and incubated in a primary antibody, rabbit anti-mouse P2X4R polyclonal antibody (1:1000, Abcam). The complete information of all antibodies were included in the Additional file 1. The next day, membranes were incubated in a horseradish peroxidase-conjugated anti-rabbit secondary antibody (1:9000, ZSGB-BIO, China) at 37 °C for 1 h. Immunoblots were then probed with a BeyoECL Plus kit (Beyotime, China) and visualized with an imaging system (Fusion, Germany). β-Actin (1:9000, Proteintech, China) was used as a loading control to normalize protein levels.

### Immunohistochemistry and quantification

For c-Fos detection, mice were treated as described above with 5-BDBD or vehicle, and tissue was collected 2 h after the last NTG injection. Mice were anesthetized with 10% chloral hydrate (0.1 ml/10 g) and perfused intracardially with 30 ml of ice-cold 0.9% saline and subsequently perfused with 50 ml ice-cold 4% paraformaldehyde (PFA)/0.1 M PBS (pH 7.4). The whole brain was collected and post-fixed in 4% PFA at 4 °C for 18 h. The medullary segment containing the TNC between + 1 and − 3 mm from the obex was removed. Tissues were immersed in 20% and 30% sucrose in turn. Samples were then frozen, and transverse sections were cut at 10 μm thickness (Leica). For c-Fos staining, sections were incubated in 0.3% H_2_O_2_ for 30 min and blocked with 10% normal goat serum in PBST (10% NRST) for 1 h, followed by incubation with anti-c-Fos antibody at 4 °C (1:500, 48 h, Abcam, USA). Sections were incubated for 1 h with a goat anti-rabbit secondary antibody and incubated with an avidin-biotin-complex using a kit (ZSGB-BIO, China). C-Fos primary antibody staining was visualized with DAB.

The TNC region was determined based on morphological appearance under light microscopy using the Mouse Brain Atlas as a reference [17]. Images were observed in a ZEISS Axio Imager A2 microscope under × 10 and × 20 objective lens. Expression of c-Fos was quantified by averaging 6–10 sections containing both sides of the TNC (throughout rostrocaudal extent) per mouse, (*n* = 7 mice/group). Image analysis was performed by Imagepro-Plus 6.0 software. The c-Fos-positive cells were scored as those with obvious specific nuclear staining. The laminae I–V of TNC were determined manually as area of interest using a rectangular tool. There was no significant difference in the area of TNC across experimental groups (see Additional file 2). All evaluations were performed by an observer blind to the experimental groups.

### Immunofluorescence staining and counting

For Iba-1 and P2X4R detection, tissues were collected at 12 h after the last NTG/saline injection. But considering the short half-life of CGRP, tissues were taken at 2 h after last NTG/saline injection for CGRP immunofluorescence staining. The sections were blocked by incubation in 10% normal donkey serum or goat serum at room temperature for 30 min. Sections were incubated with goat anti-mouse Iba-1 polyclonal antibody (1:400, Abcam), rabbit anti-mouse P2X4R antibody (1:80, Abcam), or mouse anti-mouse CGRP antibody (1:100, Santa Cruze) overnight in 4 °C, respectively. The next day, sections were incubated with species-specific fluorophore-labeled secondary antibodies (1:400, Abbkine) for 2 h at room temperature. Nuclei were counter stained with 4′,6-diamidino-2-phenylinodole (DAPI) at 37 °C for 5 min. Sections were viewed under a confocal laser scanning fluorescence microscope (ZEISS, Germany).

The TNC area on the section images was determined as previously described. Subsequently, we calculated the ratio of cross-sectional area immunoreactive for Iba1 in the TNC area and estimated the number of Iba1-IR cells per 1 mm^2^ of the TNC at × 10 or × 20 magnification using an image analysis software (Image-Pro Plus 6.2, Media Cybernetics). Optical density (OD) values were taken to indicate the quantities of CGRP and calculated by correcting the intensity of stained areas for the intensity of the background. The two sides of the TNC from six sections per animal (*n* = 6 mice/group) were included in the analysis.

### Statistical analysis

Data are expressed as mean ± SD. All statistical analyses were performed by GraphPad Prism version 6.0 (Graph Pad Software Inc., San Diego, CA). All data were tested for normality using the Kolmogorov-Smirnov (K-S) normality tests and considered normally distributed data. *F* test or Bartlett’s test were used to compare variances. Behavioral results were analyzed using a two-way RM ANOVA, with drug and time as factors. In this case, all groups were compared to responses on day 1, and to the VEH-VEH group. For Iba-1 immunofluorescence and P2X4R qRT-PCR experiments, data were analyzed using unpaired *t* tests. If the variances were significantly different, unpaired *t* test with Welch’s correction was used. The expression of P2X4R and CGRP and the number of c-Fos-positive nuclei were analyzed using one-way ANOVA followed by Tukey’s multiple comparison test, and the variance difference was not significant. A significance level of *p* < 0.05 was used.

## Results

### Effect of recurrent NTG stimulation on microglia response in the TNC

**Fig. 1:**
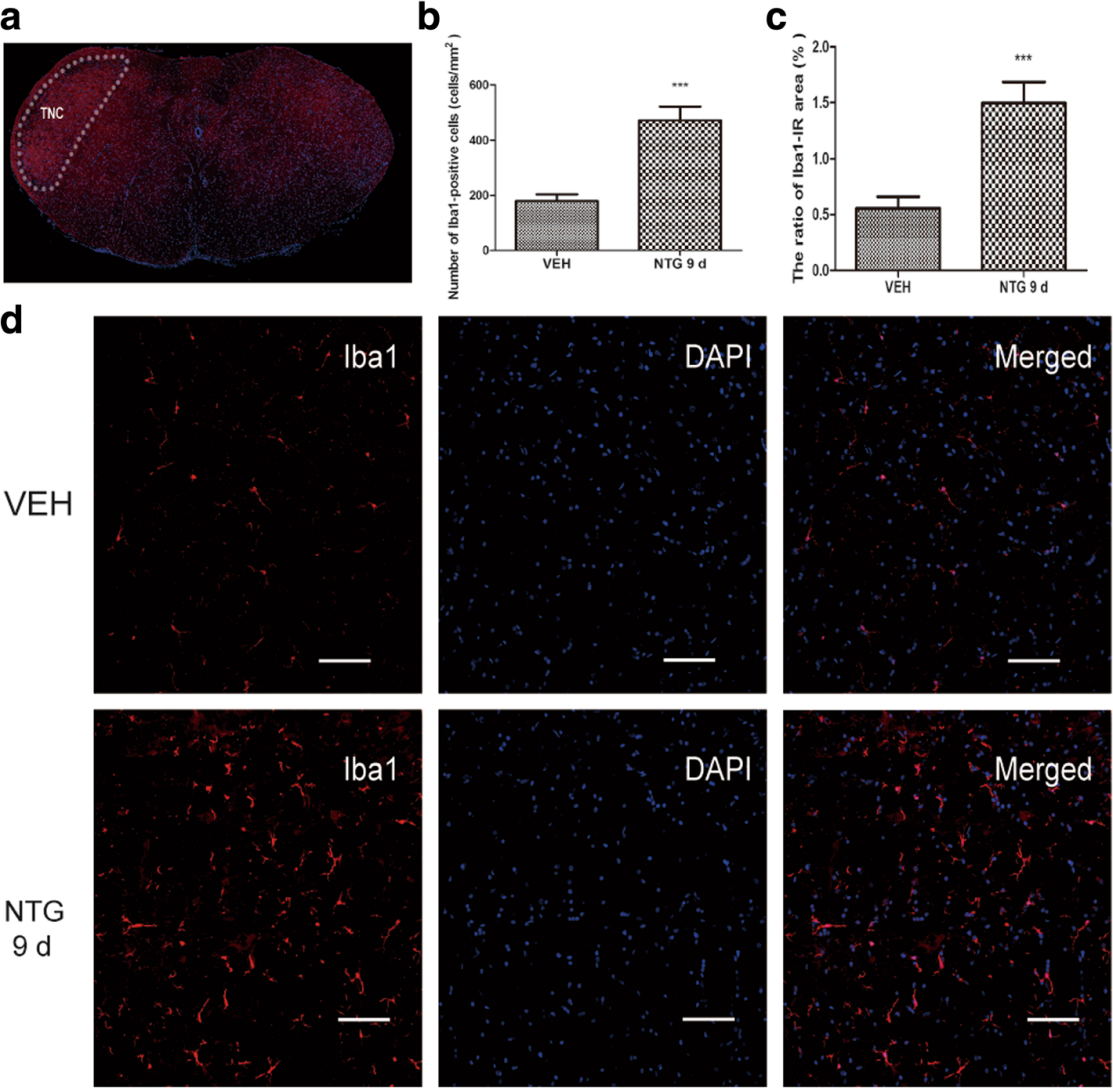
Effect of recurrent NTG stimulation on microglia response in the TNC. **a** The white dotted line frame indicates the TNC regions. **b** Quantitative analysis of the number of Iba1-IR-positive cells. **c** The ratio of area immunoreactive for Iba1 to the total area of the ipsilateral TNC in the vehicle group (VEH) and the NTG 9d group (NTG intermittent injection for 9 days). Unpaired *t* test, *n* = 6/group. ***p < 0.001. **d** Immunostaining of the TNC for Iba1 in VEH (the upper row) and the NTG 9d group (the lower row); nuclear staining was performed with DAPI (the middle column). Scar bar, 100 μm

To study the role of NTG on microglia response, we performed a qualitative immunohistochemical assessment of Iba1 (microglial specific marker) in the TNC. As shown in Fig. 1, Iba1-IR was observed sparsely in the TNC in the vehicle group (VEH). However, after recurrent administration of NTG for 9 days (NTG 9d), the number of Iba1-labeled cells (179.4 ± 24.3/mm^2^) was significantly higher than that in the vehicle group (472.6 ± 48.8/mm^2^, *p* < 0.05) (Fig. 1b). In addition, marked hypertrophy of Iba1-IR cells was observed in the NTG 9d group. Morphologically, the majority of the hypertrophic microglia appeared to be ramified microglia, and only a few ameboid microglia were identified (Fig. 1d). Quantitatively, the ratio of the cross-sectional area of Iba1-IR cells to the TNC area was 0.56 ± 0.1% in the vehicle group and 1.50 ± 0.2% in the NTG 9d group (Fig. 1c). There was a significant increase in the NTG 9d group compared to that in the vehicle group (*p* < 0.05).

**Fig. 2:**
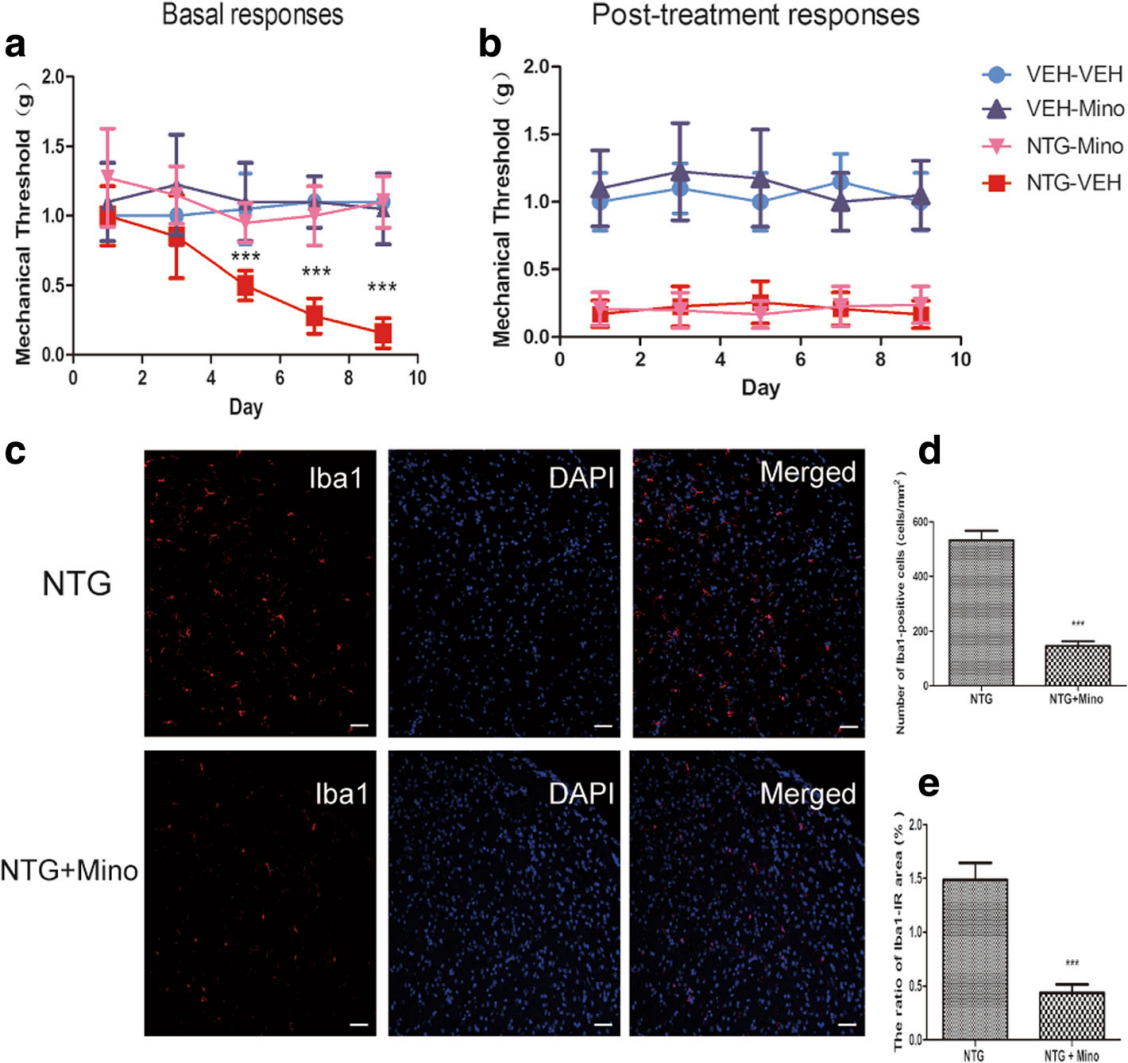
Effect of minocycline on NTG-induced mechanical hypersensitivity and microglia response. Prior to NTG/saline administration, mice were treated with vehicle or minocycline (Mino, 30 mg/kg, i.p.) every other day for 9 days. **a** Chronic treatment with minocycline significantly altered basal hypersensitivity. *p* < 0.01 for drug, time, and interaction. Two-way RM ANOVA and Bonferroni post hoc analysis. *n* = 8/group. ***p < 0.001 compared to the VEH-VEH group. **b** Post-treatment responses were assessed 2 h following NTG administration. Minocycline did not prevent acute NTG-induced hyperalgesia. *p* < 0.001 effect of drug, but no significant effect of time or interaction, two-way RM ANOVA. *n* = 8/group. **c** Immunostaining of the TNC for Iba1 in the NTG group and the NTG + Mino group on day 9; scale bar, 50 μm. **d** Quantitative analysis of the number of Iba1-positive cells. **e** The ratio of area immunoreactive for Iba1 to the total area of the ipsilateral TNC. Unpaired *t* test, *n* = 6/group. ***p < 0.001

We speculated that microglial activation correlated with the NTG-induced pain hypersensitivity. Therefore, we intraperitoneally injected an inhibitor of microglial activation, minocycline (30 mg/kg). In agreement with previous findings, repetitive intermittent NTG injection (NTG-VEH) produced a significant time-dependent chronic basal mechanical hyperalgesia as assessed by testing prior to each administration of NTG (Fig. 2a). In addition, every NTG administration evoked significant acute mechanical hyperalgesia on each test day as assessed by testing 2 h after NTG injection (Fig. 2b). Minocycline (NTG-Mino) significantly decreased the basal pain hypersensitivity but did not alter post-treatment hyperalgesia induce by NTG. Only minocycline administration (VEH-Mino) did not provoke any significant change in the mechanical threshold.

Compared with the NTG group (NTG), minocycline (NTG + Mino) reduced the number of Iba1-labeled cells on the ninth day (532.9 ± 34.6/mm^2^ vs 146.5 ± 16.7/mm^2^, *p* < 0.05). Meanwhile, minocycline also reduced the ratio of the cross-sectional area of Iba1-IR cells to the TNC area (1.49 ± 0.2% vs 0.44 ± 0.1%, *p* < 0.05) (Fig. 2c–e). These results indicated that migraine-associated pain evoked by NTG may depend on microglia activation.

### Recurrent NTG injection increased the expression levels of the P2X4R

**Fig. 3:**
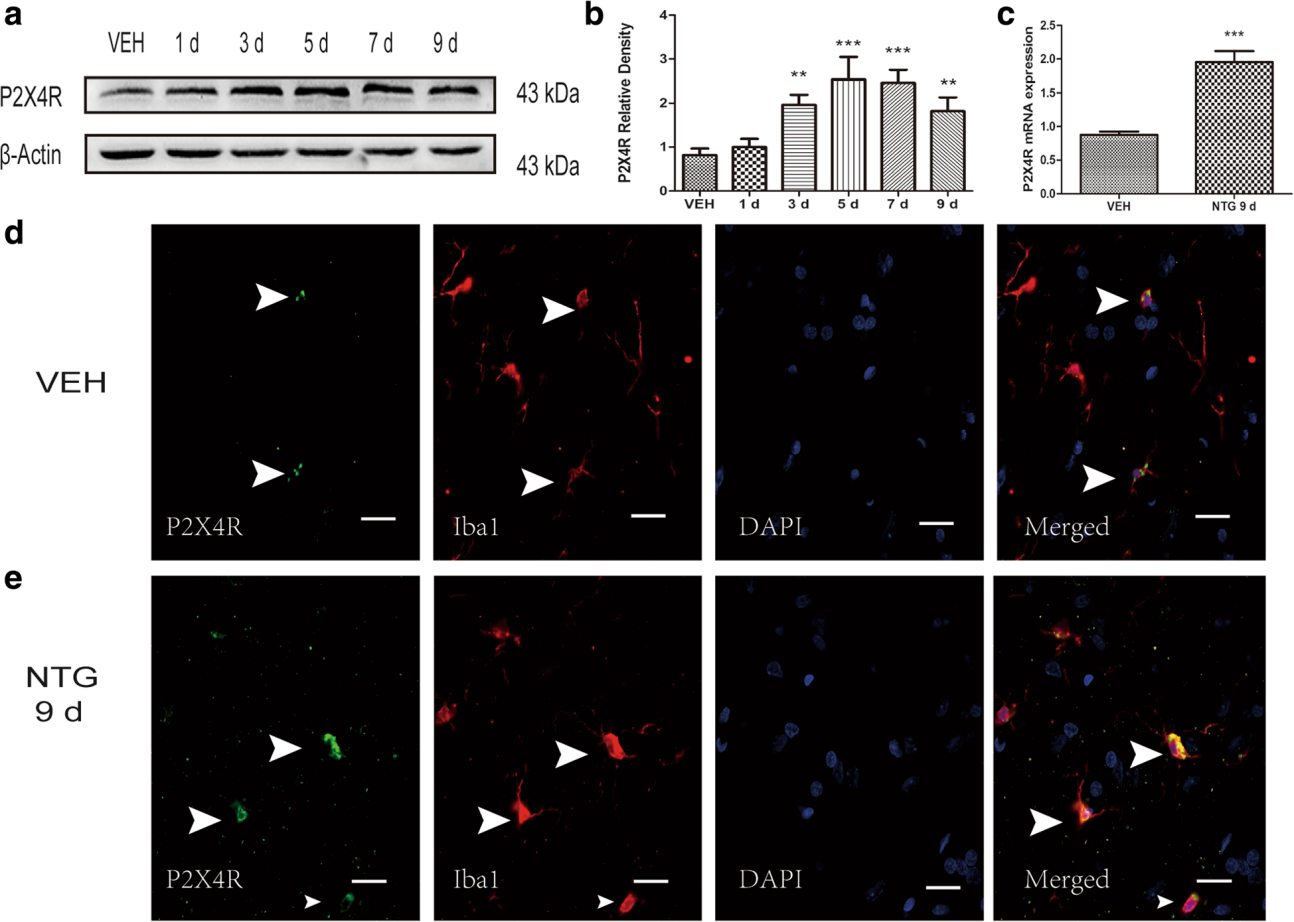
Expression profile of P2X4R after chronic intermittent administration of NTG. **a**, **b** Western blot assay for the protein expression of P2X4R on different days. One-way ANOVA and Tukey’s multiple comparison test. *n* = 5/group, **p < 0.01, ***p < 0.001, versus the vehicle group (VEH). **c** qRT-PCR analysis for the mRNA expression of P2X4R between the vehicle group and the NTG 9d group (NTG intermittent injection for 9 days). Unpaired *t* test, *n* = 6/group. ***p < 0.001. **d**, **e** Double immunofluorescence labeling of P2X4R (green) and Iba1 (red) in VEH and NTG 9d groups. Most P2X4R-positive cells are double-labeled (yellow) with Iba1. Scale bars, 20 μm

We examined the level of P2X4R expression in the TNC and found that P2X4R mRNA and protein levels increased markedly after recurrent NTG stimulation (Fig. 3a–c); the increase in P2X4R protein was detected as early as day 3. On the last day of NTG injection, the expression of P2X4R was still higher, which is in line with recurrent NTG-induced basal hyperalgesia. To identify the cell type of expressing P2X4R, we performed double immunofluorescence labeling for P2X4R and Iba1 (Fig. 3d, e). We found that almost all P2X4R-positive cells were double-labeled with Iba1, indicating that P2X4R was expressed in microglia, as shown in previous research [18].

### P2X4R antagonist 5-BDBD prevented NTG-induced mechanical hypersensitivity

**Fig. 4:**
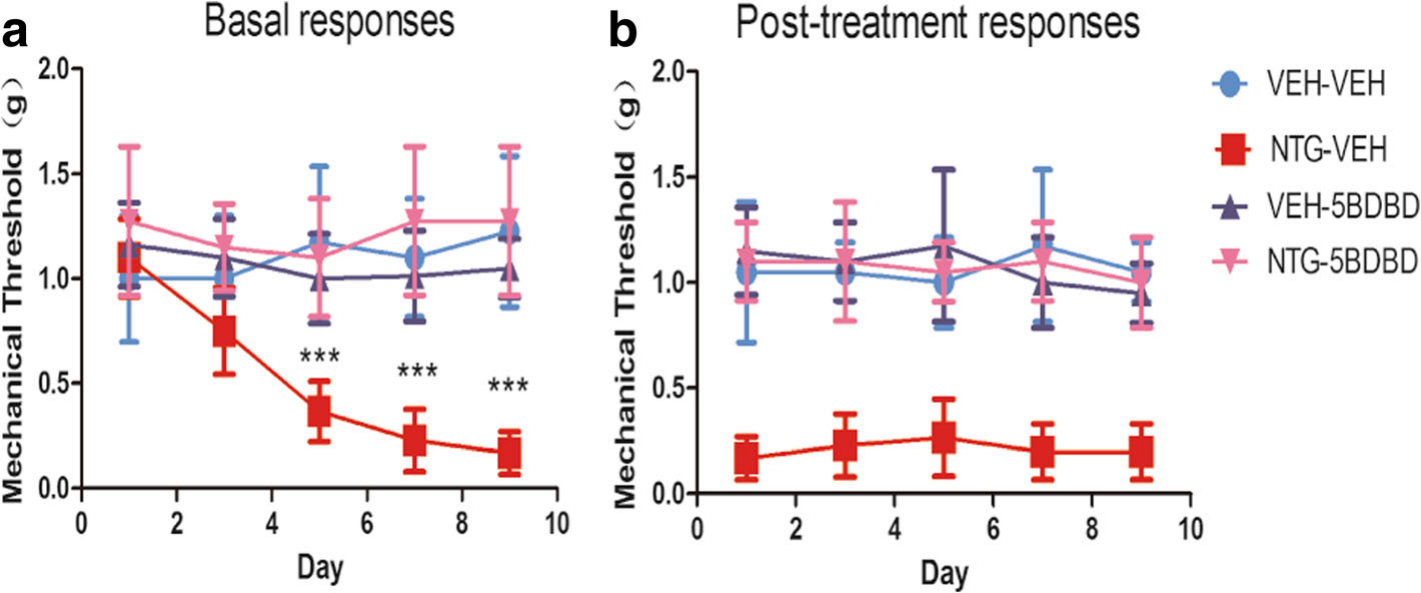
Chronic treatment with 5-BDBD (P2X4R antagonists) inhibited NTG-induced basal and acute hyperalgesia. Prior to NTG/saline administration, mice were treated with vehicle or 5-BDBD (28 mg/kg, i.p.) every other day for 9 days. **a** Basal hyperalgesia induced by recurrent NTG injection was completely blocked by 5-BDBD. *p* < 0.01 for drug, time, and interaction; two-way RM ANOVA and Bonferroni post hoc analysis; *n* = 8/group. ***p < 0.001 compared to the VEH-VEH group. **b** Every NTG injection evoked acute hyperalgesia, which was also blocked by 5-BDBD. *p* < 0.01 for drug, no significant effect of time or interaction. Two-way RM ANOVA, *n* = 8/group

Because we found that P2X4R expression was markedly upregulated in the TNC after NTG injection, we predicted that suppressing the function of P2X4R could prevent NTG-induced hypersensitivity. Mice were treated with the P2X4R antagonist 5-BDBD (28 mg/kg) or vehicle prior to injection of NTG or saline. We observed that chronic treatment with 5-BDBD (NTG + 5BDBD) completely blocked the basal hypersensitivity (Fig. 4a) and the acute NTG-induced hyperalgesia (Fig. 4b). Only 5-BDBD administration (VEH-5BDBD) did not provoke any significant change in mechanical threshold. These results indicated that P2X4R may be the key receptor participating in NTG-induced hyperalgesia.

### 5-BDBD reduced NTG-induced c-Fos expression in the TNC

**Fig. 5:**
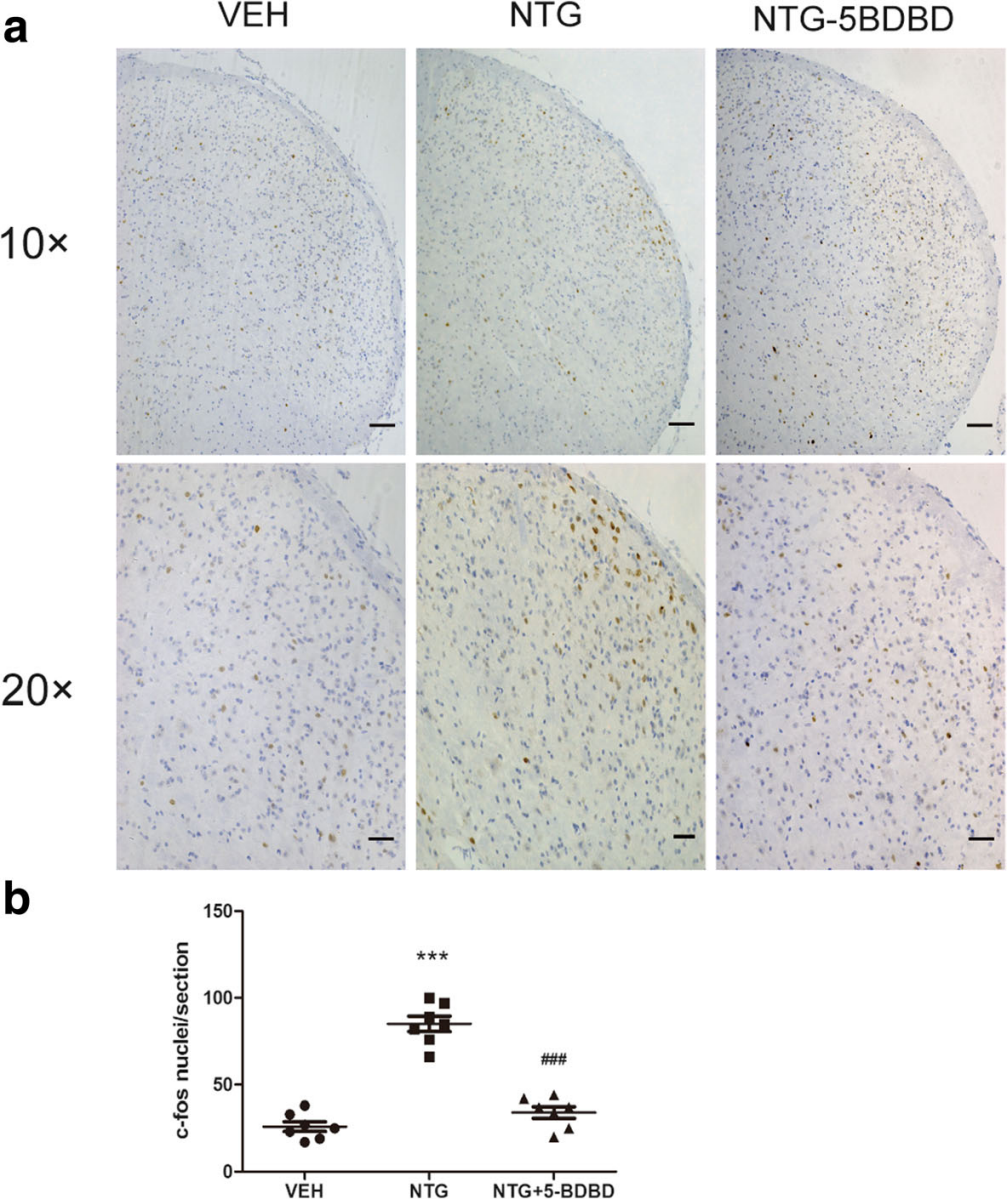
Change in c-Fos immunoreactivity in the TNC. **a** Representative images of c-Fos immunoreactivity in the VEH, NTG, and NTG + 5BDBD groups under × 10 objective lens (the upper row) and × 20 objective lens (the lower row). **b** Quantification of average number of c-Fos particles revealed that NTG significantly increased c-Fos expression in the TNC. Pretreatment with 5-BDBD inhibited NTG-induced c-Fos expression. One-way ANOVA and Tukey’s multiple comparison test. ***p < 0.001 compared to VEH, ^###^*p* < 0.001 compared to NTG group. *n* = 7/group

c-Fos has been extensively used as a reliable marker for the activation of nociceptive neurons after noxious stimulation. In line with previous studies [19], NTG evoked c-Fos expression in the superficial layer of the TNC, which may underly the NTG-induced mechanical hypersensitivity (Fig. 5a). Compared with the vehicle group (VEH), the number of c-Fos-IR cells was significantly increased after recurrent NTG stimulation (NTG) (26.1 ± 7.5 vs 84.9 ± 11.8 cells/section, *p* < 0.001). Treatment with 5-BDBD (NTG + 5BDBD) elicited a reduction in c-Fos compared with the NTG group (34.1 ± 8.7 vs 84.9 ± 11.8 cells/section, *p* < 0.001) (Fig. 5b). These results supported the notion that P2X4R might activate the central region of trigeminovascular system to potentiate central sensitization.

### Effect of 5-BDBD on CGRP protein expression in the TNC

**Fig. 6:**
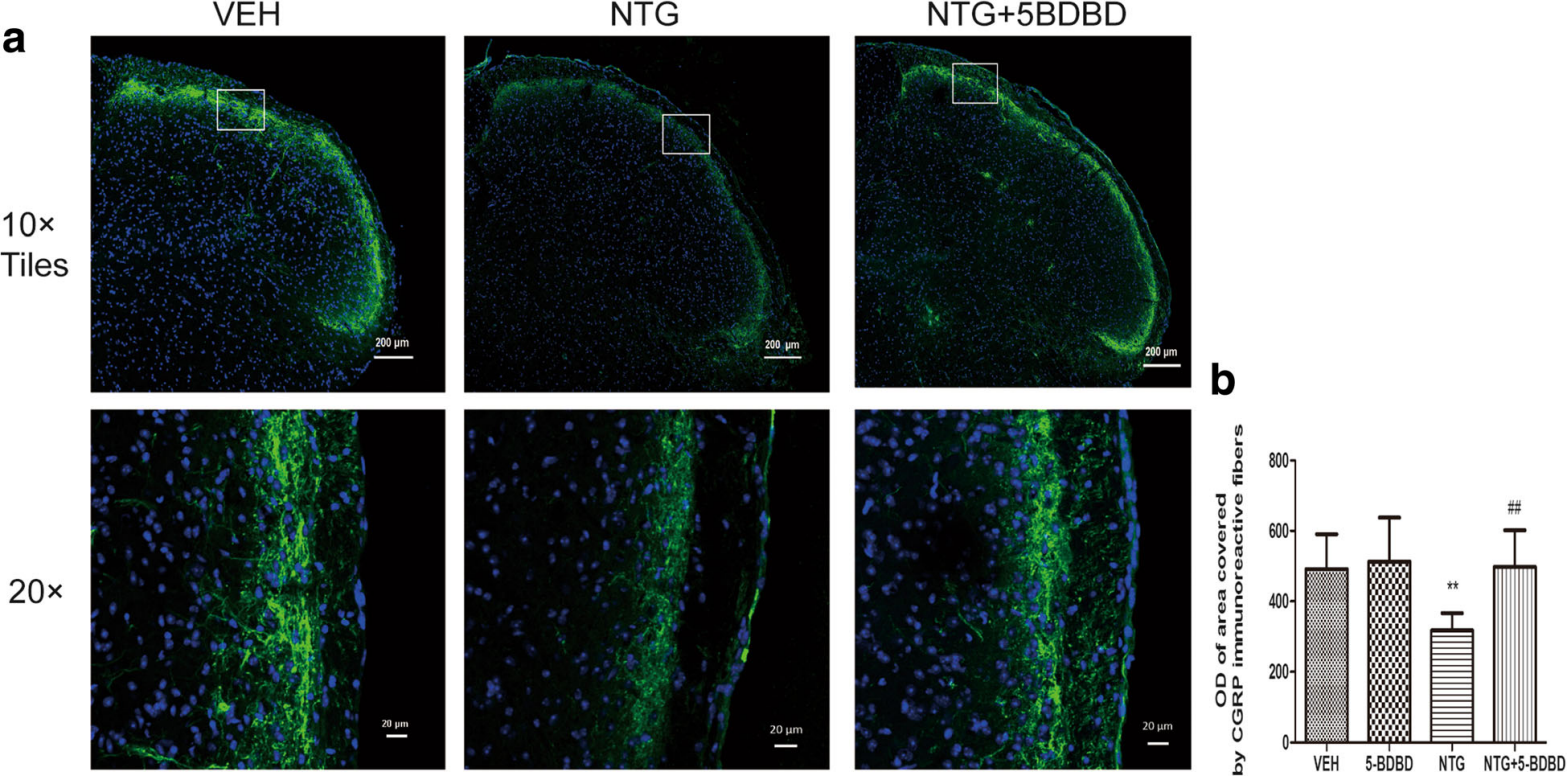
Change in CGRP immunoreactivity in the TNC. **a** Representative photos of CGRP-immunoreactive staining in the TNC of the VEH, NTG, and NTG + 5-BDBD groups under × 10 objective lens (the upper row) or and × 20 objective lens (the lower row). The white rectangle frame indicates the representative anatomical site observed under × 20objective lens. **b** Compared to the VEH group, the OD of area covered by CGRP-immunoreactive fibers was decreased in the NTG group. Pretreatment with 5-BDBD attenuated the decrease of CGRP immunopositivity. The OD of staining fibers was not significantly different at the vehicle group and NTG + 5-BDBD group. One-way ANOVA and Tukey’s multiple comparison test. *n* = 6/group. **p < 0.01 compared to the VEH, ^##^*p* < 0.01 compared to the NTG group

CGRP, an important mediator of migraine pathogenesis, plays a pivotal role in central sensitization in the TNC. NTG induces a hyperalgesia condition associated with CGRP released from central terminals of TG neurons in the brainstem. After repeated NTG injection, the OD of CGRP-immunoreactive fibers was decreased in the superficial layers of TNC than that in the vehicle group (*p* < 0.001, Fig. 6a, b). The immunopositivity decrease was attenuated by pretreatment with 5-BDBD (*p* < 0.01). These results indicated that P2X4R may be involved in cross-talk mechanism between TNC neurons and microglia.

## Discussion

The aim of this study was to investigate whether microglia P2X4R in the TNC contributes to central sensitization in CM. Following chronic intermittent NTG administration for 9 days, an acute hyperalgesia and prolonged basal hypersensitivity were induced in mice, as manifested by a decrease in the mechanical threshold in response to von Frey filaments. In these recurrent NTG injection mice, we found enhancement of Iba1-immunoreactivity and P2X4R expression in the TNC. Pretreatment with minocycline alleviated chronic basal hyperalgesia and microglia activation, but not acute NTG-induced hyperalgesia. The P2X4R inhibitor, 5-BDBD, completely prevented the acute and chronic mechanical hyperalgesia. Recurrent NTG-induced c-Fos expression and CGRP release in the TNC were also attenuated after 5-BDBD treatment. All of these results suggest that aberrant microglia activation and P2X4R expression may be the critical component regulating migraine chronification.

### Animal model of chronic migraine

We used chronic intermittent NTG injection (10 mg/kg, i.p.) to build a CM animal model, described recently by Pradhan et al. [13]. NTG is a widely used migraine trigger in human clinical experiments [20]. Acute NTG-induced thermal and mechanical hyperalgesia in rodents has been used as an animal model for migraine. Moreover, chronic administration of NTG to mice resulted in not only acute mechanical hyperalgesia after each exposure but also sustained and progressive decrease in the basal mechanical threshold of hind paws. These results are consistent with the clinical observations of CM patients in whom hyperalgesia or allodynia may occur in both cephalic region and extracephalic region, such as the neck and forearms. The appearance of cutaneous allodynia following NTG likely reflects the development of central sensitization, which may be mediated through abnormal neuronal excitability within the trigeminal nucleus caudalis (TNC) and thalamus. Some researches determined the mechanical sensitivity of periorbital region and hind paws. Similar to the hind paws, NTG treatment also induces the periorbital hyperalgesia [21]. So in our study, we only tested the mechanical sensitivity of animal hind paws. This chronic basal hyperalgesia persists for days following cessation of NTG administration. Further, repeated administration of NTG has also been shown to produce photophobia, hypoactivity, and facial grimace behaviors [22]. Therefore, this is a reliable animal model for studying chronic migraine-associated pain.

In our study, recurrent NTG administration produced a significant basal sensitization, which was first observed after two NTG injections (i.e., day 5). In addition, on each test day, NTG also evoked significant acute mechanical hyperalgesia.

### Chronic migraine and microglia

Most research on central sensitization mainly focuses on neuronal function, ignoring the potential contribution of gliocytes. Extensive studies about animal models of neuropathic pain have indicated that spinal glial activation, especially microglia, and the subsequent secretion of mediators have an important role in initiating or maintaining an aberrant hyperalgesia state [23].

Recently, some evidences have indicated that altered microglia function may be involved in the pathophysiological mechanisms of migraine. It is known that recurrent CSD is the upstream neural signal change in CM [24]. A recent study indicated that multiple CSD caused a marked enlargement of microglia [5] and release of microglia cytokine, such as IL-1β and TNF-α. These cytokines also affected the threshold and amplitude of CSD [25–27]. In addition, Cui Y and colleagues also found in a PET study that microglia were activated in the rat brain as a consequence of CSD stimulation [28].

In addition, Nathan Fried used another CM animal model that was built with repeated IS stimulation of dura mater. Then, the author indicated that no microglia activation was observed during the episodic stage (only two IS stimulations), but microglia activation was increased in the TNC during the chronic stage (total of ten IS stimulations). Importantly, minocycline treatment could inhibit trigeminal sensitivity after recurrent IS stimulation [7].

Our results showed that after recurrent NTG injection, the number of Iba1-IR cells was increased in the TNC. Minocycline significantly attenuated the NTG-induced basal mechanical hyperalgesia and immunoreactive staining for Iba1, but it did not alter acute hyperalgesia. Thus, inhibition of microglial activation can be used as a potential preventive therapy rather than an acute analgesic. However, in addition to its effects on microglia, minocycline has been shown to have broad effects on immune cells, such as astrocytes, oligodendrocytes, and macrophages. As such, data generated with minocycline need to be interpreted with caution. We cannot rule out that minocycline may affect other immune cells to decrease NTG-induced hypersensitivity. In addition, the cellular mechanism that underlies the crosstalk between microglia and neurons of the TNC needs further study.

### Chronic migraine and purinergic signal

Although *N*-methyl-D-aspartate (NMDA) and non-NMDA receptors are thought to be fundamental mechanisms underlying central sensitization, many studies suggest that purinergic receptor may be involved [29]. In the purinergic hypothesis for migraine, ATP has been implicated in migraine via stimulating cerebral vasodilatation and primary afferent nerve terminals located in the cerebral microvasculature.

A substantial number of studies have revealed an increasing microglia-specific P2X4R expression in the spinal cord in diverse animal models of neuropathic pain. In our experiments, a similar change in P2X4R expression in the TNC was found. Furthermore, the P2X4R-specific inhibitor, 5-BDBD, inhibits P2X4R-mediated Ca2+ signals and inward ion currents in vitro and is known to permeate the blood-brain barrier [30]. We found that 5-BDBD, unlike minocycline, effectively prevented both basal hyperalgesia and acute NTG-induced hyperalgesia. P2X4R is also expressed in other areas of the brain; therefore, we cannot exclude the possibility that 5-BDBD acts on other migraine-related regions including the periaqueductal gray (PAG), sensory cortical region, or even satellite glial cells (SGCs) of the trigeminal ganglion (TG) [31]. In addition, minocycline does not affect the activation of SGCs in the TG [32, 33]. Therefore, minocycline and 5-BDBD may play different roles in NTG-associated hyperalgesia.

### P2X4R, c-Fos, and CGRP

C-Fos, the protein of the protooncogene c-fos, has been widely used as a marker for neuronal activation. Most treatments inhibiting c-Fos similarly affect pain-related behaviors. In addition, c-Fos has been implicated in central sensitization via transcriptional regulation of prodynorphin [34]. Studies using different animal models of migraine (including injection of NTG, IS, or CSD stimulation) all evoked an increase expression of c-Fos within the TNC, especially in laminae I and II [35]. In our study, a chronic NTG model significantly induced c-Fos expression in the superficial lamina of the TNC, where the first-order trigeminal neurons make synaptic contact with the second-order neurons. In addition, 5-BDBD alleviated NTG-induced c-Fos expression, which may underlie the reduction in the pain threshold.

CGRP, as a key neuropeptide of the trigeminal system, is implicated in the peripheral and central sensitization of migraine. During migraine attacks, the levels of CGRP in patient serum, saliva, and cerebrospinal fluid are elevated. In the animal model of migraine, NTG injection results in an increase of CGRP in the blood, dura mater, TG, and TNC [36]. However, some previous studies found that NTG treatment induced a decrease in the area occupied by CGRP-immunoreactive fibers and in CGRP protein expression by western blot in the TNC [37, 38]. These results indicated that this is a probably consequence of an increase release of CGRP from storage vesicles in the central terminals of trigeminal afferents. The majority of CGRP is synthesized in cell bodies of TG. Then, CGRP protein is transported in vesicles along the nerve fibers to the nerve endings in the TNC or solitary tract nucleus [39]. In our study, 2 h after NTG treatment was the time of sampling for determining the CGRP protein levels; these periods might be insufficient for the depleted CGRP to be resynthesized and to be transported to TNC. So we found a reduction of CGRP staining after repeated NTG injection. In addition, NO synthesis or cGMP activation may be involved in the mechanism of NTG-induced CGRP release [40].

In our studies, CGRP-immunoreactive fibers were mainly distributed in the superficial of TNC, which are associated primarily with processing nociceptive information. 5-BDBD pretreatment mitigated the NTG-induced CGRP immunoreactivity changes in the TNC, modulating the trigeminal system activation. The cellular mechanism underlying the P2X4R-induced CGRP release may be related to brain-derived neurotrophic factor (BDNF). Previous studies demonstrated that BDNF is released from ATP-stimulated microglia via the P2X4R signaling pathway in vitro and vivo [41]. In addition, BDNF, acting via trkB receptors, can regulate the expression and release of CGRP from sensory neurons [42].

## Conclusion

Chronic migraine occurs with central sensitization of trigeminal sensory pathways that involve microglial activation and increased expression of P2X4R. Inhibition of microglia may be effective in preventing migraine chronification but be ineffective in aborting an acute migraine attack. In addition, we speculated that P2X4R may be implicated in microglia-neuronal signaling in the TNC. Inhibition of P2X4R function might be a potential therapeutic option for CM.

## Additional files

Additional file 1: Complete information for antibodies in the research. (DOCX 16 kb)

Additional file 2: **Figure S1.** Western blot for CGRP in the vehicle group on day 1. **Figure S2.** Western Blot for P2X4R in the vehicle group on day 1. **Figure S3.** There was no difference in the size of TNC areas. (DOCX 6150 kb)

## Abbreviations

ATP: Adenosine triphosphate
BDNF: Brain-derived neurotrophic factor
CGRP: Calcitonin gene-related peptide
CM: Chronic migraine
CSD: Cortical spreading depression
IS: Inflammatory soup
NMDA: *N*-Methyl-d-aspartate
OD: Optical density
PAG: Periaqueductal gray
qRT-PCR: Quantitative real-time polymerase chain reaction
SGC: Satellite glial cells
TG: Trigeminal ganglion
TNC: Trigeminal nucleus caudalis
