# Supplementary material for: Microglia P2X4 receptor contributes to central sensitization following recurrent nitroglycerin stimulation
Source: J Neuroinflammation. 2018 Aug 30;15:245. doi: 10.1186/s12974-018-1285-3 (PMC6117935; doi:10.1186/s12974-018-1285-3)
Supplement: Supplementary file 1 — Complete information for antibodies in the research. (DOCX 16 kb) [file 12974_2018_1285_MOESM1_ESM.docx]

**Additional file 1**. **Complete information for antibodies in the research.**

CGRP antibody was purchased at Santa Cruze Biotechnology Company (Catalog: sc-57053, Lot number:#A0816), which is a mouse monoclonal antibody raised against a CGRP of rat origin. P2X4 receptor rabbit polyclonal antibody was purchased at Abcam Company (Catalog: ab50319, Lot number: GR 253826-1). Synthetic peptide: KKYKYVEDYEQGLSGEMNQ, by an N-terminal Cysteine residue linker, corresponding to amino acids 370-388 of Rat P2RX4. This antibody stains a main bands of 43 kDa molecular weight on Western Blot. Because of polyclonal antibody, in addition to the 43 kDa band there are also several other bands. But they were not obvious compared with 43 Kda band. Iba1 goat polyclonal antibody was purchased at Abcam Company (Catalog: ab5076, Lot number: GR 3175346-1). Synthetic peptide corresponding to Human Iba1 aa 135-147 (C terminal). Accession Number (s): NP_116573.1; NP_001614.3. Sequence: C-TGPPAKKAISELP. C-Fos mouse monoclonal antibody was purchased at Abcam Company (Catalog: ab208942, Lot number: GR 269424-2). Recombinant full length protein corresponding to Human c-Fos aa 1-380. purified from E. coli.
