# Supplementary material for: Microglia P2X4 receptor contributes to central sensitization following recurrent nitroglycerin stimulation
Source: J Neuroinflammation. 2018 Aug 30;15:245. doi: 10.1186/s12974-018-1285-3 (PMC6117935; doi:10.1186/s12974-018-1285-3)
Supplement: Supplementary file 2 — Figure S1. Western blot for CGRP in the vehicle group on day 1. Figure S2. Western Blot for P2X4R in the vehicle group on day 1. Figure S3. There was no difference in the size of TNC areas. (DOCX 6150 kb) [file 12974_2018_1285_MOESM2_ESM.docx]

**Additional file 2**.


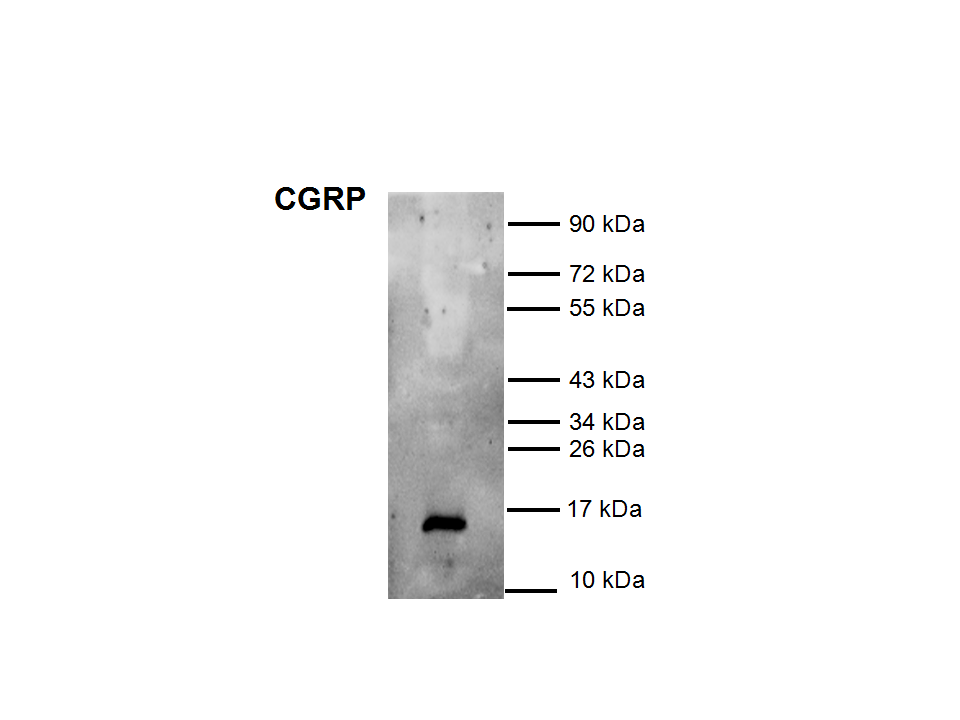


***Figure S1.* Western blot for CGRP in the vehicle group on day 1**. Predicted band size: 15 kDa.

**
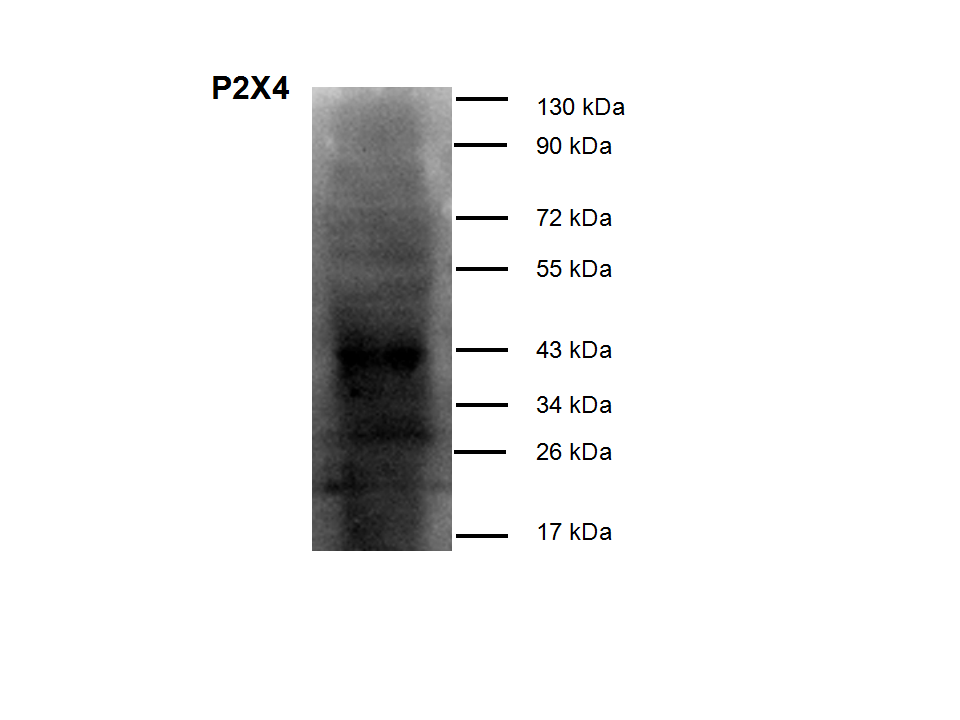
**

***Figure S2.*** **Western Blot for P2X4R in the vehicle group on day 1**. Predicted band size: 43 kDa.


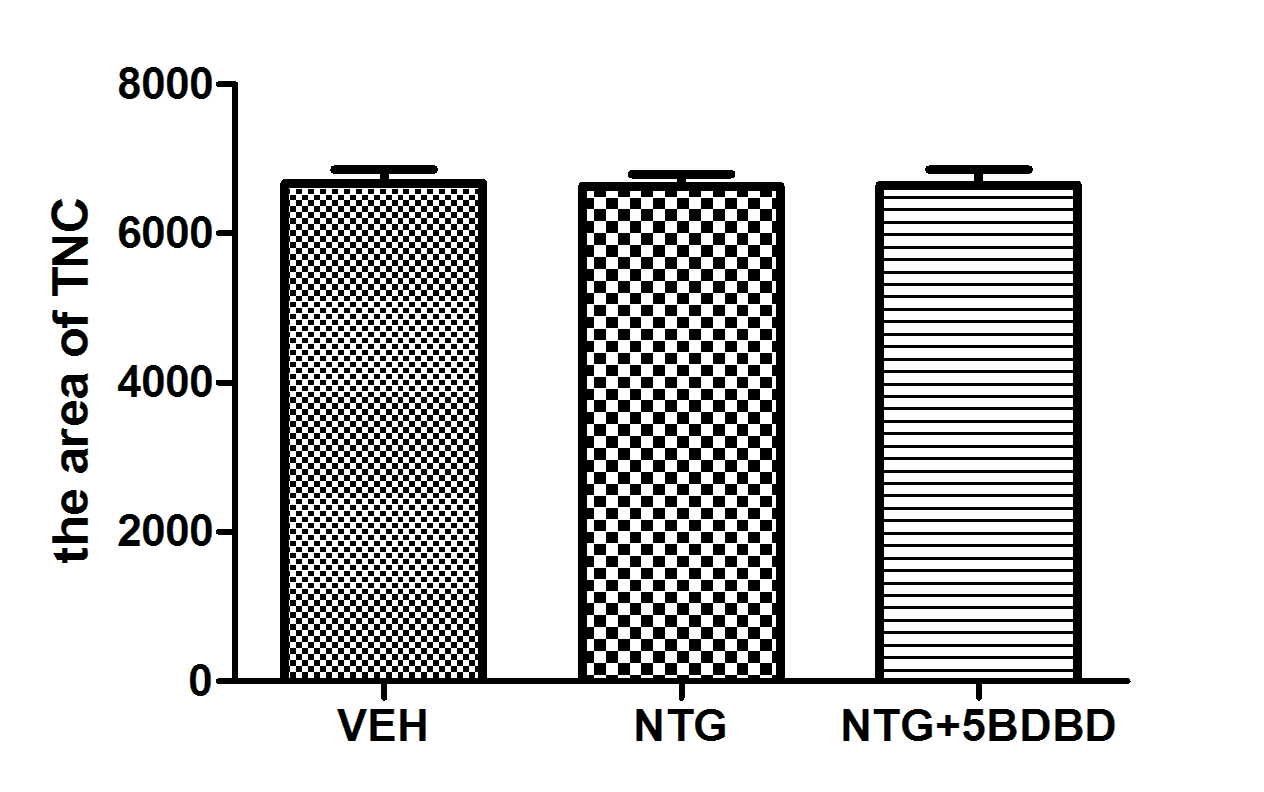


***Figure S3.* There was no difference in the size of TNC areas.** For c-Fos detection, mice were randomly divided into three groups: VEH, NTG and NTG+5-BDBD group. The laminae I–Ⅴ of TNC were determined manually as area of interest using a rectangular tool. The size of TNC area were 6674.66 ± 513.62 (um2) in the VEH group, 6639.65 ± 447.74 (um2) in the NTG group, and 6653.14 ± 571.95 (um2) in the NTG+5-BDBD group. No significant difference was found across experimental groups (one-way ANOVA and Tukey’s Multiple comparison test, n=6/groups).
